# Bidirectional Regulation of AdpA_ch_ in Controlling the Expression of *scnRI* and *scnRII* in the Natamycin Biosynthesis of *Streptomyces chattanoogensis* L10

**DOI:** 10.3389/fmicb.2018.00316

**Published:** 2018-03-02

**Authors:** Pin Yu, Qing-Ting Bu, Yi-Li Tang, Xu-Ming Mao, Yong-Quan Li

**Affiliations:** ^1^Institute of Pharmaceutical Biotechnology, Zhejiang University, Hangzhou, China; ^2^Zhejiang Provincial Key Laboratory for Microbial Biochemistry and Metabolic Engineering, Hangzhou, China; ^3^College of Life Sciences, Zhejiang University, Hangzhou, China

**Keywords:** bidirectional regulation, AdpA, natamycin biosynthesis, *Streptomyces chattanoogensis* L10, pathway-specific gene

## Abstract

AdpA, an AraC/XylS family protein, had been proved as a key regulator for secondary metabolism and morphological differentiation in *Streptomyces griseus*. Here, we identify AdpA_ch_, an ortholog of AdpA, as a “higher level” pleiotropic regulator of natamycin biosynthesis with bidirectional regulatory ability in *Streptomyces chattanoogensis* L10. DNase I footprinting revealed six AdpA_ch_-binding sites in the *scnRI*–*scnRII* intergenic region. Further analysis using the *xylE* reporter gene fused to the *scnRI*–*scnRII* intergenic region of mutated binding sites demonstrated that the expression of *scnRI* and *scnRII* was under the control of AdpA_ch_. AdpA_ch_ showed a bi-stable regulatory ability where it firstly binds to the Site C and Site D to activate the transcription of the two pathway-specific genes, *scnRI* and *scnRII*, and then binds to other sites where it acts as an inhibitor. When Site A and Site F were mutated *in vivo*, the production of natamycin was increased by 21% and 25%, respectively. These findings indicated an autoregulatory mechanism where AdpA_ch_ serves as a master switch with bidirectional regulation for natamycin biosynthesis.

## Introduction

The secondary metabolic process in *Streptomyces* is regulated by a complex regulatory network involving pathway-specific, pleiotropic, and global regulators which respond to a variety of physiological and environmental condition alterations (van Wezel and McDowall, 2011; Liu et al., 2013). The best characterized is the A-factor regulatory cascade in which AdpA is the most important transcriptional factor for the secondary metabolism (Horinouchi, 2002; Ohnishi et al., 2005). In early culture stages, the transcription of *adpA* in *Streptomyces griseus* is repressed by ArpA, the receptor protein for A-factor (Onaka and Horinouchi, 1997). When A-factor reaches a critical concentration, it binds to ArpA and confers the conformational change of ArpA (Ohnishi et al., 1999). This results in dissociation of ArpA from the *adpA* promoter, in turn switching on the expression of *adpA* (Ohnishi et al., 1999). The induced AdpA then activates the transcription of various genes related to secondary metabolism such as *strR*, the pathway-specific regulatory genes for streptomycin in *S. griseus* (Retzlaff and Distler, 1995; Tomono et al., 2005).

AdpA is a member of the AraC/XylS family proteins (Gallegos et al., 1997). It has been suggested to form a dimer through the N-terminal portion which belong to the ThiJ/PfpI/DJ-1 family (Yamazaki et al., 2004; Ohnishi et al., 2005). To date, a number of AdpA orthologs have been described as having essential roles in the secondary metabolism in many *Streptomyces* species, such as *Streptomyces lividans* (Guyet et al., 2013), *Streptomyces coelicolor* A3(2) (Takano et al., 2001; Nguyen et al., 2003), *Streptomyces ansochromogenes* (Pan et al., 2009), *Streptomyces avermitilis* (Komatsu et al., 2010), *Streptomyces hygroscopicus* 5008 (Tan et al., 2015), and *Streptomyces clavuligerus* (López-García et al., 2010).

Typically, AdpA is regarded as an activator for downstream regulated genes, except itself which is proved to be negatively auto-regulated by binding to its own promoter region (Kato et al., 2005b; Hara et al., 2009). The molecular mechanism of transcriptional activation begins as a dimer of AdpA binds to the target sites with consensus sequences which then recruit RNA polymerase to the promoter for transcriptional initiation (Yamazaki et al., 2004; Kato et al., 2005a). For different target genes, AdpA showed a different number of binding sites in the promoter regions. For example, there are two AdpA-binding sites in the promoter of *strR* (Tomono et al., 2005), whereas there are three AdpA-binding sites for regulation of *ssgA* (Yamazaki et al., 2003a). However, the precise regulation mechanism how the AdpA binds to multiple sites to activate transcription has not been experimentally determined. Based on the importance of AdpA in the biosynthesis of the secondary metabolism, it is necessary to elucidate details of its regulatory mechanisms.

Natamycin, an antifungal polyene macrolide antibiotic, is synthesized by a type I polyketide synthase gene cluster. Previous analysis of the gene cluster of natamycin in *Streptomyces chattanoogensis* L10 revealed the existence of 17 open-reading frames, including two pathway-specific genes, *scnRI* and *scnRII* (Du et al., 2011a). These two genes showed high sequence identity to *pimR* and *pimM* of *Streptomyces natalensis*, respectively (Antón et al., 2007; Santos-Aberturas et al., 2012). Gene disruption of *scnRI* resulted in a large decrease in the expression of biosynthetic genes, indicating its role as a pivotal activator for the biosynthesis of natamycin (Du et al., 2011a). *scnRII*, adjacent but divergently transcribed transcriptional regulatory genes, was shown to act as a second positive regulator for natamycin production (Du et al., 2009). We also had proved that AdpA_ch_ controls the production of natamycin, but the detailed relationship among AdpA_ch_, ScnRI, and ScnRII had not been well characterized (Du et al., 2011a).

Here, we reveal the sophisticated regulatory characteristics of AdpA_ch_ in the natamycin biosynthesis of *S. chattanoogensis* L10. AdpA_ch_ acts as a “higher level” pleiotropic regulator for transcription of the two divergently transcribed pathway-specific genes, *scnRI* and *scnRII*. In this regulatory process, AdpA_ch_ shows a bi-stable regulatory ability, where it firstly acts as an activator, then a repressor. Moreover, natamycin production was enhanced by mutating the AdpA_ch_-binding sites which had an inhibitory effect. This work not only advances the understanding of detailed regulatory mechanism of AdpA, but also provides a potential target for the enhancement of other antibiotic production levels by manipulating the regulatory network.

## Results

### AdpA_ch_ Identified as a “Higher Level” Pleiotropic Regulator for Natamycin Biosynthesis

In our previous study, the biosynthetic gene cluster of natamycin has been cloned and characterized in *S. chattanoogensis* L10. Within this there are two divergently transcribed genes, *scnRI* and *scnRII*, encoding proteins that resemble pathway-specific regulators (Du et al., 2009, 2011a). Although the functions of these two regulators have been well characterized, an important question remains as to whether there are multiple levels of control in the biosynthesis of natamycin. Based on our previous study that AdpA_ch_ affected the transcription of these two pathway-specific genes (Du et al., 2011a), we speculated that AdpA_ch_ may act as a “higher level” pleiotropic regulator for regulating the natamycin biosynthesis.

**FIGURE 1:**
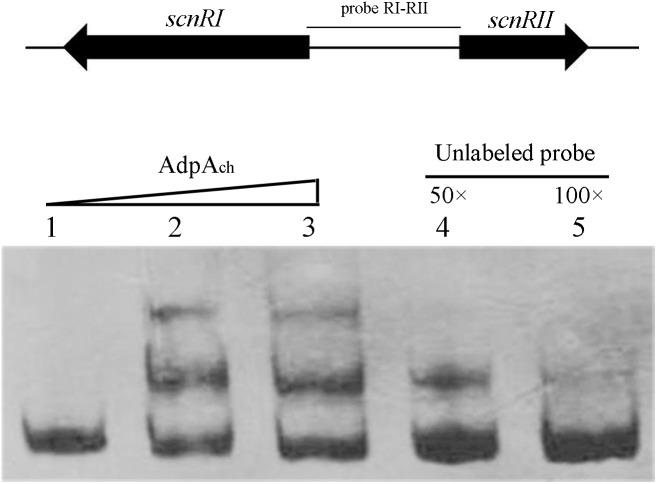
AdpA_ch_ binds to the DNA sequence of the intergenic promoter region between *scnRI* and *scnRII*. Lanes 1–3, DNA probe with AdpA_ch_ protein 0, 50, and 100 pM, respectively. Lanes 4 and 5, 50- and 100-fold excess of unlabeled specific PCR product was added into binding reactions.

To test this hypothesis, electrophoretic mobility shift assays (EMSAs) were applied. As shown in **Figure 1**, retardation was readily detected upon the addition of 50 pM AdpA_ch_ with the probe RI–RII, while the addition of 50- to 100-fold excess of unlabeled specific PCR product reduced the proportion of the labeled promoter-containing fragment (**Figure 1**). These data clearly demonstrate that AdpA_ch_ could specifically bind to the *scnRI*–*scnRII* intergenic region and could control the expression of these two pathway-specific genes.

### DNase I Footprinting Assay Reveals Six AdpA_ch_-Binding Sites in the *scnRI*–*scnRII* Intergenic Region

**FIGURE 2:**
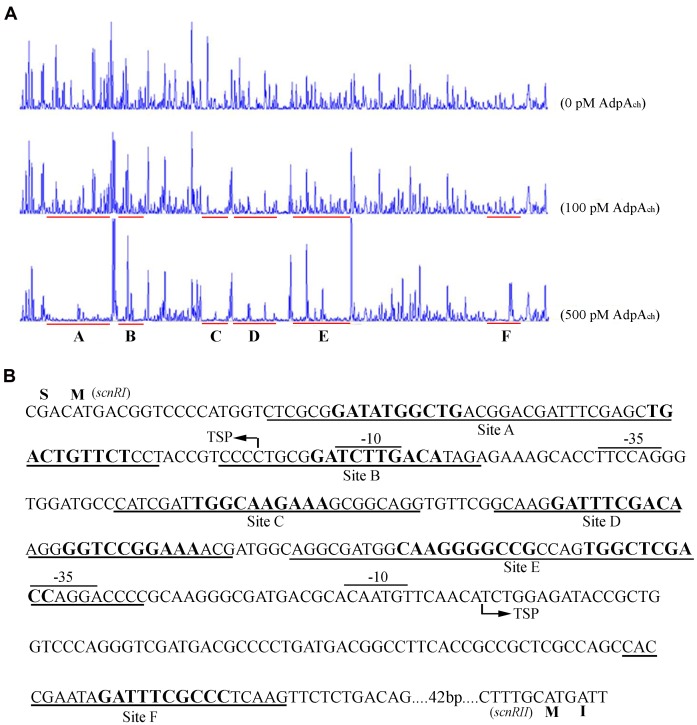
DNase I footprinting assay for determination of the AdpA_ch_-binding sites. **(A)** A 5′-FAM-labeled probe p*RI-RII* was used in the DNase I footprinting assay with 0, 100, and 500 pM purified AdpA_ch_, respectively. The protected regions are underlined. **(B)** Nucleotide sequences of the *scnRI*–*scnRII* intergenic region showing the predicted AdpA_ch_-binding sites. The TSS is marked by a bent arrow, the AdpA_ch_-binding sites are underlined, and the –10 and –35 regions are overlined.

To identify the exact DNA sequences that AdpA_ch_ protected in the *scnRI*–*scnRII* intergenic region, DNase I footprinting assays, in absence or presence of purified recombinant AdpA_ch_, were performed. In our previous studies, we had determined the transcription start site (TSS) of the two pathway-specific genes, *scnRI* and *scnRII* (Du et al., 2011a). As seen in **Figure 2A**, at a lower AdpA_ch_ protein concentration of 100 pM, the DNA strands of the *scnRI*–*scnRII* intergenic region showed two protected regions, Site C and Site D, extending from positions -69 to -44 and -106 to -74 relative to the TSS of *scnRI*. When increasing the protein concentration to 500 pM, another four protected regions (Sites A, B, E, and F) were observed. With respect to the *scnRI* TSS, the AdpA_ch_-binding Site A locates at positions +8 to +54, Site B at positions -20 to +2, Site E at positions -161 to -114, and Site F at positions -283 to -259 (**Figure 2B**). The six AdpA_ch_-binding sites were spread over the *scnRI*–*scnRII* intergenic region. Notably, Site A was located downstream of the *scnRI* TSS, while Site B overlapped the -10 region of the *scnRI* promoter. Site F was located downstream of the *scnRII* TSS, and Site E overlapped the -35 region of the *scnRII* promoter. This data suggest that AdpA_ch_ might have a negative regulatory ability for the expression of these two pathway-specific genes. Additionally, the results from the DNase I footprinting assay also reveal that AdpA_ch_ may have higher affinity to Site C and Site D than to the others.

### The Consensus AdpA_ch_-Binding Sequence in the AdpA_ch_-Binding Sites

**FIGURE 3:**
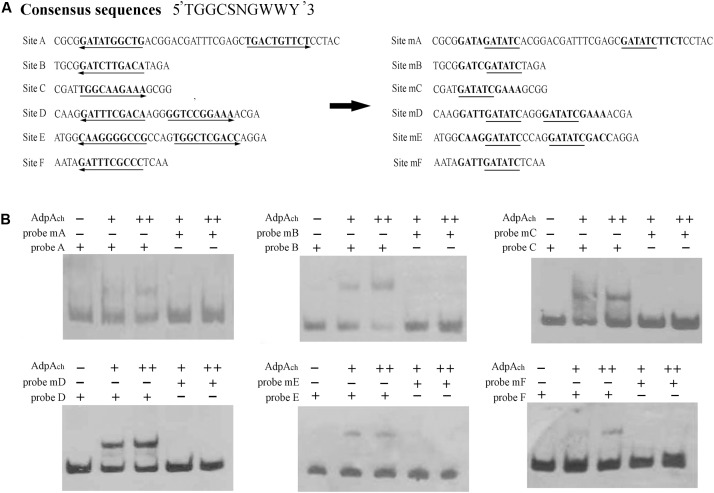
Mutational analysis of the AdpA_ch_-binding sites. **(A)** Mutations introduced in the six putative AdpA_ch_-binding sites. The predicted AdpA_ch_-binding consensus sequences are in bold, and these consensus sequences are changed with an *EcoRI* site indicated with underlines. **(B)** EMSAs for determination of AdpA_ch_ binding to mutated sequences. Probes A–F contained the fragment of Sites A–F as shown in **A**, respectively. Probes mA–mF contained the fragment of corresponding mutated sites. The amounts of AdpA_ch_ protein used were 50 and 100 pM.

The orthologs of AdpA_ch_ identified in *S. griseus* and *S. coelicolor* have been reported to have the consensus binding sequence, 5-TGGCSNGWWY-3 (S: G or C; W: A or T; Y: T or C; N: any nucleotide) (Yamazaki et al., 2004). After alignment of these six protected regions, we also found that there were highly conserved AdpA_ch_-binding sequences in each binding site (**Figure 3A**). To further study the roles of these consensus sequences in the AdpA_ch_-binding ability, EMSAs were carried out using the probes containing either the sequences of wild-type (wt) binding sites or the mutated sites (**Figure 3A**). As shown in **Figure 3B**, no binding shift was detected for the mutated sites A–F when compared with their corresponding wt targets. Taken together, these data demonstrated that AdpA_ch_ indeed has six binding sites in the *scnRI*–*scnRII* intergenic region and the consensus sequence is essential for the binding activity of AdpA_ch_.

### AdpA_ch_ Has Differing Affinities for Different Binding Sites

**FIGURE 4:**
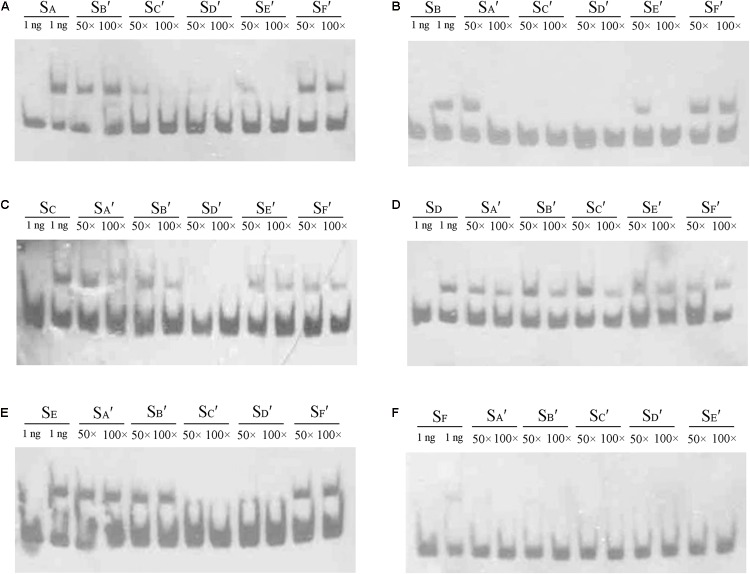
Comparison of the relative affinity of AdpA_ch_ with different binding sites. Labeled probes S_A_, S_B_, S_C_, S_D_, S_E_, and S_F_ contained the fragment of Sites A–F as shown in **Figure 3A**, respectively. Probes S_A′_, S_B′_, S_C′_, S_D′_, S_E′_, and S_F′_ also contained the fragment of Sites A–F as shown in **Figure 3A**, respectively, but they are unlabeled. The amount of AdpA_ch_ protein used was 100 pM.

In the DNase I footprinting analysis, Site C and Site D were occupied with a lower concentration of AdpA_ch_ than the other sites. This suggests that there may be affinity differences for AdpA_ch_ between the six binding sites. To test this possibility, competitive EMSAs with 50- to 100-fold excess of unlabeled fragments of six AdpA_ch_-binding sites were used to compete with each labeled fragment. As shown in **Figure 4A**, 100-fold excess of unlabeled S_B_′ (Site B) and S_F_′ (Site F) could not completely abolish AdpA_ch_ complex formation with the labeled probe S_A_ (Site A). However, the same amount of unlabeled S_C_′ (Site C), S_D_′ (Site D), and S_E_′ (Site E) outcompeted the labeled probe S_A_. This result indicated that AdpA_ch_ binds to Site A more tightly than Site B and Site F, but less tightly than Site C, Site D, and Site E. Following this way, we could conclude that Site B has less affinity for AdpA_ch_ than others, except for Site F (**Figure 4B**), which was the weakest affinity among the six binding sites (**Figure 4F**), and Site D was the strongest affinity of these six sites (**Figure 4D**). The affinity of Site E for AdpA_ch_ was between that of Site C and Site A (**Figures 4A,C,E**). Therefore, we determined the affinity of AdpA_ch_ to different binding sites in the following order: Site D > Site C > Site E > Site A > Site B > Site F.

### Promoter-Probe Assays of the AdpA_ch_-Binding Sites in the *scnRI*–*scnRII* Intergenic Region

**FIGURE 5:**
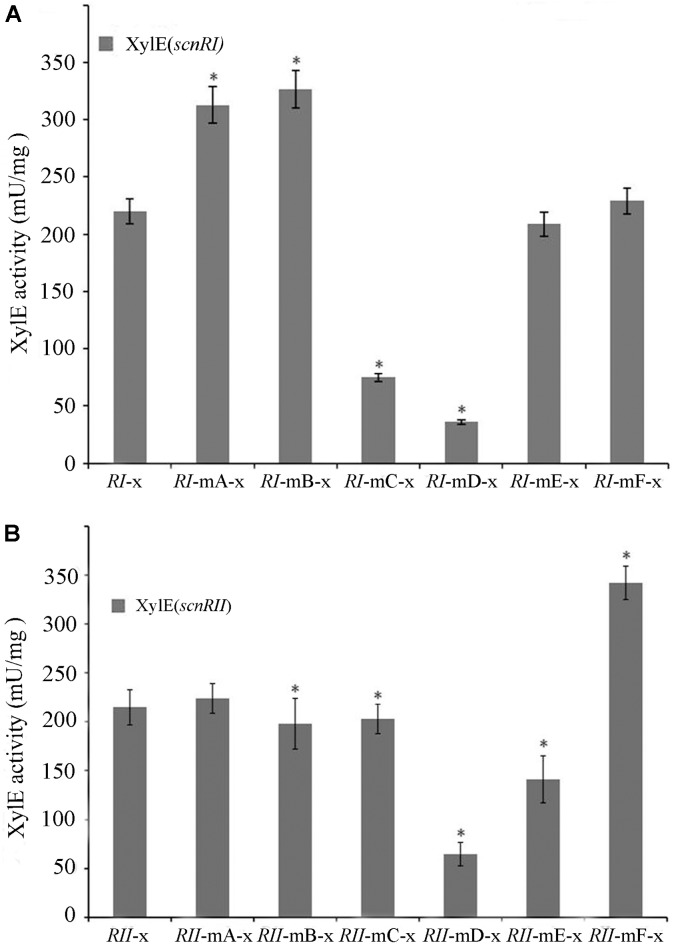
Promoter activities of *scnRI* **(A)** and *scnRII* **(B)** with the effect of mutations in the AdpA_ch_-binding sites. The strains were grown in YEME medium for 24 h, and catechol dioxygenase activity was calculated as the change of catechol quantity (mmol) per minute. Error bars correspond to the standard error of the mean of four culture replicates. ^∗^ Indicates significant differences between promoter mutants and promoter wt (*P* < 0.05).

The binding sites of AdpA_ch_ in the *scnRI*–*scnRII* intergenic region were adjacent to either the *scnRI* or the *scnRII* start codon. This raised the possibility that this intergenic region might harbor a bidirectional promoter allowing AdpA_ch_ to regulate transcriptions of the divergently transcribed flanking genes, *scnRI* and *scnRII* (**Figure 2B**). To investigate the promoter activities of the two pathway-specific genes with each of the AdpA_ch_-binding sites, we used the promoter-probe plasmid pIJ8601 carrying the *xylE* gene, encoding catechol 2,3-dioxygenase, as the reporter. As shown in **Figure 5A**, the transcriptional profiles of *scnRI* were severely decreased when the AdpA_ch_-binding Site C and Site D were mutated. Conversely, its transcriptional activity was increased when Site A and Site B were mutated and remained almost unchanged when Site E and Site F were mutated. For the promoter activity of *scnRII*, we did not detect any consistent differences when Sites A, B, and C were mutated, but mutation in the Sites D and E resulted in a large decreases of up to 70 and 40%, respectively, compared to those of the wt. The mutation in Site F resulted in a statistically significant increase (**Figure 5B**). These findings indicated that expressions of *scnRI* and *scnRII* are both under the control of AdpA_ch_, which has a completely different regulatory ability (activation or inhibition) when binding to different binding sites.

### Effect of Mutated AdpA_ch_-Binding Sites On Natamycin Production *in Vivo*

**FIGURE 6:**
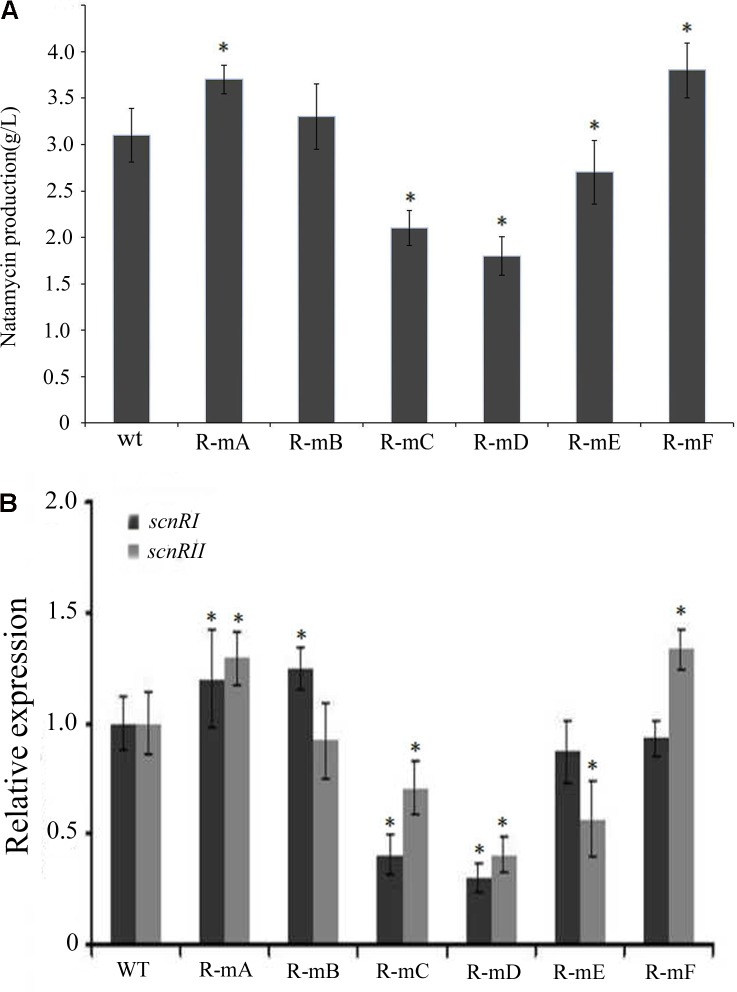
**(A)** The effect of mutated AdpA_ch_-binding sites on the natamycin production *in vivo*. The strains were grown in YEME medium for 96 h. Vertical error bars correspond to the standard error of the mean of four replicated cultures. **(B)** Real-time RT-PCR analysis of the *scnRI* and *scnRII* transcript in the wt strain and mutated AdpA_ch_-binding sites strain. The expression level of *scnRI and scnRII* is presented relative to the wt sample from 24 h, which was arbitrarily assigned a value of 1. The transcription of *hrdB* was assayed as an internal control. Error bars were calculated by measuring the standard deviation among three replicates of each sample. ^∗^ Indicates significant differences between AdpA_ch_-binding site mutants and wt (*P* < 0.05).

There have been some reports where effects upon DNA-binding sites were found *in vitro* that failed to be exhibited *in vivo*. In order to test this possibility and reveal the function of the six AdpA_ch_-binding sites in natamycin biosynthesis *in vivo*, a series of mutants were constructed as described in Experimental procedures. As shown in **Figure 6A**, compared to the WT strain, the level of natamycin production in the R-mA (mutation in Site A) and R-mF (mutation in Site F) had increased by 21 and 25%, respectively. However, the constructed strains of R-mC (mutation in Site C), R-mD (mutation in Site D), and R-mE (mutation in Site E) showed up to 31, 42 and 15% reductions, respectively. The natamycin production of R-mB (mutation in Site B) mutant exhibited almost no change. This finding indicated that the AdpA_ch_-binding Sites A and F play negative roles for natamycin biosynthesis, while the functions of the Sites C, D, and E were positive. Quantitative real-time PCR (qRT-PCR) analysis showed that the promoting effect of site mutation on natamycin production was due to alteration of the pathway-specific genes at the transcriptional level (**Figure 6B**).

## Discussion

*Streptomyces* spp. have developed complicated mechanisms to adapt to altered circumstances (Santos-Beneit et al., 2009; Yu et al., 2012). Among these mechanisms, the multiple levels of regulation in controlling the expression of the genes responsible for the formation of the secondary metabolism are drawing increased attention. In this study, we focused on the regulatory network of natamycin biosynthesis in *S. chattanoogensis* L10, an industrial strain for natamycin production. In our previous study, we determined that gamma-butyrolactones (GBLs) serve as quorum-sensing signaling molecules for activating natamycin production in *S. chattanoogensis* L10 (Du et al., 2011b), and ScnRII acts as a positive regulator by directly binding to the promoters of natamycin biosynthetic genes (Du et al., 2009) where ScnRI acts as a positive regulator for the transcription of *scnRII* (Du et al., 2011a). However, the deletion of *scnRI* did not result in a complete halt of the transcription of *scnRII* (our unpublished data). This is quite different from the function of PimR in *S. natalensis* where the deletion of *pimR* almost completely destroys the transcription of *pimM* (Antón et al., 2004; Santos-Aberturas et al., 2012). As the regulation of antibiotic biosynthesis involves numerous transcription factors (McKenzie and Nodwell, 2007; van Wezel and McDowall, 2011), participation of other regulator(s) is possible, in the regulation of *scnRII*.

With AdpA_ch_ being able to regulate the expression of both of the pathway-specific genes, *scnRI* and *scnRII*, it provides a possible explanation that there is a coordinate regulation in controlling expression of *scnRII* by AdpA_ch_ and ScnRI. This regulatory pattern may occur in following steps. Firstly, AdpA_ch_ binds to the *scnRI*–*scnRII* intergenic region and activates both transcription of *scnRI* and *scnRII*. Then ScnRI also binds to the *scnRI*–*scnRII* intergenic region which, in turn, promotes the transcriptional level of *scnRII*. However, these two genes were not completely controlled by AdpA_ch_. Trace expression of *scnRI* was observed in the *adpA_ch_* mutant, and then ScnRI would promote the transcription of *scnRII* (Du et al., 2011a). Notably, a certain amount of AdpA_ch_ is required for binding to the *scnRI*–*scnRII* intergenic region (∼50 pM). This is why we did not detect the shifted band with low concentration AdpA_ch_ (∼1 pM) in the binding reaction of our previous study (Du et al., 2011a).

In most cases, AdpA acts as an activator for the target genes, except for itself where it exhibits an autorepression (Kato et al., 2005b). In this study, we concluded from promoter-probe assays *in vivo* that AdpA_ch_ could not only regulate both pathway-specific genes, but also displayed completely opposite regulatory abilities in control of them. The AdpA_ch_-binding Site C and Site D were involved in activating the transcription of *scnRI*, while AdpA_ch_ binding to Sites A and B resulted in repression. For the promoter activity of *scnRII*, mutation in the Site C and Site D resulted in a decrease of transcriptional profiles, while a mutation in the Site F led to a statistically significant increase. A similar phenotype was observed in *S. ansochromogenes* where transcription of *sanG* decreased when Site I and Site V were mutated but increased when other three AdpA-L-binding sites were mutated (Pan et al., 2009). However, when combinations of binding site mutations were carried out, the promoter activities were not in accordance with our predictions. For example, mutations in both Sites E and F reduced the transcriptional level of *scnRII* (data not shown). Based on the short distances between the AdpA_ch_-binding sites which are spread over the *scnRI*–*scnRII* intergenic region, there may be complicated interactions between different AdpA_ch_ dimmers to explain this.

**FIGURE 7:**
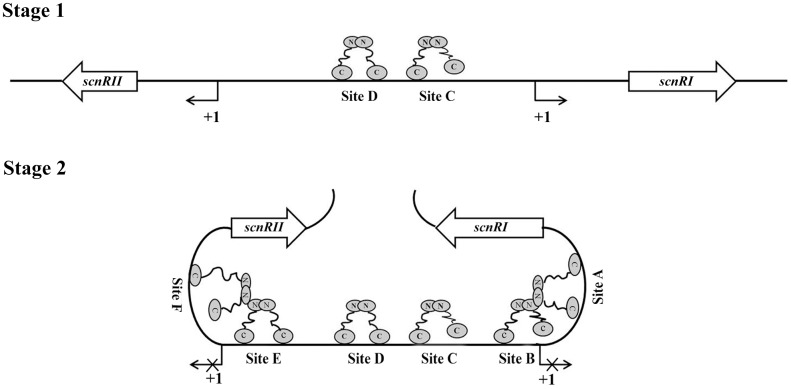
Proposed model of AdpA_ch_ regulation for *scnRI* and *scnRII* transcription. The AdpA_ch_-binding sites are spread over the *scnRI*–*scnRII* intergenic region, and the presumptive manner of AdpA_ch_-binding forms a dimmer. Stage 1: AdpA_ch_ binds to the Site C and Site D when the concentration of AdpA_ch_ is low in the early growth stage. Stage 2: When the concentration AdpA_ch_ reaches a high level, AdpA_ch_ binds to Site A, Site B, Site E, and Site F, most of which located downstream of the -35 sequences of the corresponding genes. A DNA loop may be formed via interaction between different AdpA_ch_ dimmers, thus preventing RNA polymerase from access to the promoter.

With further analysis using competitive gel shift assays, we could conclude that AdpA_ch_ binds to Sites A–F with the following affinities: Site D > Site C > Site E > Site A > Site B > Site F (**Figure 4**). These data are consistent with the footprinting assay where the regions of Site C and Site D were previously protected at a lower AdpA_ch_ protein concentration (**Figure 2A**). This gives a hint that the regulatory ability of AdpA_ch_ may occur in a growth phase-dependent manner. In the early stage, AdpA_ch_ firstly binds to the Site C and D to recruit RNA polymerase to the promoter and initiates the transcription of *scnRI* and *scnRII*. This in turn triggers natamycin production (**Figure 7**). When AdpA_ch_ is accumulated to a certain critical level, it will bind to other binding sites located near the TSS. A DNA loop may be formed via the interaction between different AdpA_ch_ dimers, thus preventing RNA polymerase from access to the promoter of the pathway-specific genes (**Figure 7**). Reduced transcription of the pathway-specific genes will result in a low rate of natamycin production.

The discovery of this bidirectional regulation of AdpA_ch_ in the control of natamycin biosynthesis reveals an artful adaptive mechanism in microbial cells. Microorganisms produce molecules with antibiotic activity and expel them into the environment, presumably enhancing their ability to compete with their neighbors (Berdy, 2005; Hopwood, 2007). However, most of these molecules are toxic to the producer (Mak et al., 2014; Moody, 2014). Mechanisms must exist to ensure that antibiotic production reaches a reasonable level. The proposed model of AdpA_ch_ in **Figure 7** may provide a fresh mechanistic insight into how *S. chattanoogensis* controls the production level of natamycin via AdpA_ch_. However, further work will be needed to prove the proposed model and the detailed mechanism of how AdpA_ch_ responds to the signal of natamycin. In all, the complicated regulatory network involving AdpA_ch_, ScnRI, and ScnRII helps advance our understanding of the molecular regulation mechanisms of antibiotic biosynthesis and provides an effective strategy to help improve yields in industrial strains.

**Table 1:** Bacterial strains and plasmids used in this work.

Strains/plasmids	Characteristics	Reference
Strains		
*E. coli* TG1	General cloning host	Novagen
*E. coli* ET12567/pUZ8002	Methylation-deficient *E. coli* for conjugation with the helper plasmid	Macneil and Klapko, 1987
*E. coli* BL21 (DE3)	A host for protein expression	Novagen
*E. coli* BW25113/pIJ790	Strain used for PCR-targeted mutagenesis	Gust et al., 2003
Wt	*S. chattanoogensis* L10 wt; natamycin producer	Du et al., 2009
*RI*-x	wt with pIJ8601-*pRI*	This study
*RI*-mA-x	wt with pIJ8601-*pRI-mA*	This study
*RI*-mB-x	wt with pIJ8601-*pRI-mB*	This study
*RI*-mC-x	wt with pIJ8601-*pRI-mC*	This study
*RI*-mD-x	wt with pIJ8601-*pRI-mD*	This study
*RI*-mE-x	wt with pIJ8601-*pRI-mE*	This study
*RI*-mF-x	wt with pIJ8601-*pRI-mF*	This study
*RII*-x	wt with pIJ8601-*pRII*	This study
*RII*-mA-x	wt with pIJ8601-*pRII-mA*	This study
*RII*-mB-x	wt with pIJ8601-*pRII-mB*	This study
*RII*-mC-x	wt with pIJ8601-*pRII-mC*	This study
*RII*-mD-x	wt with pIJ8601-*pRII-mD*	This study
*RII*-mE-x	wt with pIJ8601-*pRII-mE*	This study
*RII*-mF-x	wt with pIJ8601-*pRII-mF*	This study
R-mA	wt with mutation in Site A	This study
R-mB	wt with mutation in Site B	This study
R-mC	wt with mutation in Site C	This study
R-mD	wt with mutation in Site D	This study
R-mE	wt with mutation in Site E	This study
R-mF	wt with mutation in Site F	This study
Plasmids		
pTA2 vector	General cloning vector	TOYOBO
p-*RI-RII*	pTA2 containing the fragment of the *scnRI*–*scnRII* intergenic region	This study
pIJ8601	Streptomyces integrative shuttle vector with *xylE* reporter gene	This study
pIJ8601-*pRI*	pIJ8601 with the promoter of *scnRI*	This study
pIJ8601-*pRI-mA*	pIJ8601-*pRI* with mutation in Site A	This study
pIJ8601-*pRI-mB*	pIJ8601-*pRI* with mutation in Site B	This study
pIJ8601-*pRI-mC*	pIJ8601-*pRI* with mutation in Site C	This study
pIJ8601-*pRI-mD*	pIJ8601-*pRI* with mutation in Site D	This study
pIJ8601-*pRI-mE*	pIJ8601-*pRI* with mutation in Site E	This study
pIJ8601-*pRI-mF*	pIJ8601-*pRI* with mutation in Site F	This study
pIJ8601-*pRII*	pIJ8601 with the promoter of *scnRII*	This study
pIJ8601-*pRII-mA*	pIJ8601-*pRII* with mutation in Site A	This study
pIJ8601-*pRII-mB*	pIJ8601-*pRII* with mutation in Site B	This study
pIJ8601-*pRII-mC*	pIJ8601-*pRII* with mutation in Site C	This study
pIJ8601-*pRII-mD*	pIJ8601-*pRII* with mutation in Site D	This study
pIJ8601-*pRII-mE*	pIJ8601-*pRII* with mutation in Site E	This study
pIJ8601-*pRII-mF*	pIJ8601-*pRII* with mutation in Site F	This study

## Materials and Methods

### Media, Plasmids, Strains, and Growth Conditions

All plasmids and bacterial strains used in this study are listed in **Table 1**. General techniques for the manipulation of nucleic acids and bacterial growth were carried out according to the standard protocols as previously described (Kieser et al., 2000). *Escherichia coli* DH5α was the general cloning host. Vectors used were pSET152, pIJ8660, pTA2. *S. chattanoogensis* L10 strains were grown at 28°C on YMG agar for sporulation and at 30°C in YEME medium (3 g/l yeast extract, 3 g/l malt extract, 5 g/l tryptone, 10 g/l glucose) for natamycin production.

### Electrophoretic Mobility-Shift Assays (EMSAs)

His-AdpA_ch_, histidine-tagged protein was purified from the soluble fractions of *E. coli* BL21 (DE3) harboring the plasmids pET32a-*adpA_ch_*, as previously described (Du et al., 2011a). The Bradford reagent (Bio-Rad) was used to determine the protein concentration. For probe preparation, all primers used in this study are listed in Supplementary Table S1. The EMSA DNA probe RI–RII (517 bp) spanning the entire *scnRI*–*scnRII* intergenic region was amplified by PCR using primer pair RI–RII-F and RI–RII-R. The PCR product was then cloned into a pTA2-vector (TOYOBO) to generate the plasmid pT-*RI*–*RII*. The biotin-labeled probe RI–RII was made with 5′-biotin-labeled M13 universal primer pair using pT-*RI*–*RII* as a template by PCR amplification. The probes A (295 bp), B (281 bp), C (294 bp), D (282 bp), E (288 bp), F (284 bp), mA (295 bp), mB (281 bp), mC (294 bp), mD (282 bp), mE (288 bp), and mF (284 bp) were prepared following the above-mentioned method. In the EMSAs assay, 1 ng of the probe was incubated with varying quantities of AdpA_ch_, at 25°C for 30 min in the buffer (20 mM Tris, pH 7.5, 5% glycerol, 0.01% BSA, 50 μg ml^-1^ sheared sperm DNA). For the competition assay, 100 times of excessive un-labeled probes and non-specific DNA were added to the reaction buffer, respectively. Reactions were displayed on 5% acrylamide gels for separation in 0.5× TBE buffer. EMSA gels were then electro-blotted onto the nylon membrane and UV-fixed by UV crosslinker. Labeled DNA was detected with streptavidin-HRP and BeyoECL plus (Beyotime, China) as described by the manufacturer.

### DNase I Footprinting Assay

DNase I footprinting assay was performed as previously described (Mao et al., 2009). Firstly, AdpA_ch_ protein was ultra-filtered with YM-10 (Millipore) for 10 kD cut-off and eluted in 20 mM Tris buffer, pH 7.5. Then, FAM-labeled probe was amplified using 5′-(6-FAM)-labeled M13 universal primers from plasmid pT-*RI*-*RII*, followed by gel recovery. About 50 ng of fluorescently labeled probe was added to the reaction mixture to a final volume of 50 μl. After binding of the AdpA_ch_ protein to 5′-(6-FAM)-labeled probe (30°c, 30 min), 0.01 U of DNase I (Promega) was added for 1 min at 30°C, followed with equal volume of 100 mM EDTA to stop the reactions and extracted by phenol/chloroform. After precipitation with 40 μg of glycogen, 0.75 M ammonium acetate (NH_4_Ac), and ethanol, the digested DNA mixture was loaded into ABI 3130 DNA sequencer with Liz-500 DNA marker (MCLAB). DNA sequencing ladder was prepared according to Thermo Sequenase Dye Primer Manual Cycle Sequencing Kit (USB).

### Alterations of the Consensus Sequence for AdpA_ch_-Binding Sites

The consensus sequence of AdpA_ch_-binding sites A–F was replaced by the sequence of EcoRV restriction sequence sites using overlapping primers (Supplementary Table S1). The PCR product was then cloned into a pTA2-vector (TOYOBO). The resulted plasmids were used as template for PCR to amplify mutated probes using 5′-biotin-labeled M13 universal primers, and the binding ability was measured by EMSAs.

### Construction and Analysis of Transcriptional Fusions to the *xylE* Reporter Gene

For *xylE* fusions, the *xylE* gene was PCR amplified with the primers *xylE*-F and *xylE*-R. This fragment was digested with *Nde*I and *Not*I, and introduced into the likewise-digested pIJ8660 (Sun et al., 1999) to construct pIJ8601. To probe *scnRIp* and *scnRIIp* activities with the mutation of AdpA_ch_-binding sites, the wt and mutated promoter regions were amplified by PCR using upstream primers carrying a BamHI site listed in Supplementary Table S1. These promoter fragments were cloned into BamHI-cut pIJ8601 and transferred by conjugation into *S. chattanoogensis* L10. Plasmid-containing strains were grown on YEME medium for 24 h. Cell pellets from 1 ml culture samples were kept on ice and measured immediately. Assays of catechol 2,3-dioxygenase were performed as previously described (Kieser et al., 2000).

### Mutational Analysis of the AdpA_ch_-Binding Sites On Natamycin Biosynthesis

The 1.8 kb DNA fragment containing the sequence of *scnRI*–*scnRII* intergenic region was amplified by PCR using primers scnRI-F and scnRII-R. The resulted 1.8 kb sequence was used as template to amplify the DNA fragment for construction of mutated AdpA_ch_-binding sites *in vivo* using overlapping primers (Supplementary Table S1), then PCR product was purified and ligated into pKC1139. The resulting plasmids containing DNA fragment of mutated sites was conjugated by *E. coli* ET12567/pUZ8002 into *S. chattanoogensis* L10. The mutants were selected by replica plating for apramycin-sensitive colonies and they were used as template for PCR with primer pairs RI-RII-F and RI-RII-R. The amplified sequences were digested with EcoRV to confirm the mutants.

### Determination of Natamycin Production by HPLC Analysis

Natamycin production was confirmed by HPLC analysis with the Agilent 1100 HPLC system. HC-C_18_ column (5 μm, 4.6 by 250 mm) was used with UV detector set at 303 nm. Mobile phase and gradient elution process were as described previously (Du et al., 2009).

## Author Contributions

PY, Q-TB, and Y-LT performed the experiments. X-MM assisted with the primary data analysis. Y-QL supervised the project and revised the manuscript. All authors reviewed the manuscript.

## Conflict of Interest Statement

The authors declare that the research was conducted in the absence of any commercial or financial relationships that could be construed as a potential conflict of interest.

## References

[B1] Antón N. Santos-Aberturas J. Mendes M. V. Guerra S. M. Martín J. F. Aparicio J. F. (2007). PimM, a PAS domain positive regulator of pimaricin biosynthesis in *Streptomyces natalensis*. *Microbiology* 153 3174–3183. 10.1099/mic.0.2007/009126-0 17768260

[B2] Antón N. Vendes M. V. Martin J. F. Aparicio J. F. (2004). Identification of PimR as a positive regulator of pimaricin biosynthesis in *Streptomyces natalensis*. *J. Bacteriol.* 186 2567–2575. 10.1128/JB.186.9.2567-2575.2004 15090496 PMC387814

[B3] Berdy J. (2005). Bioactive microbial metabolites : a personal view. *J. Antibiot.* 58 1–26. 10.1038/ja.2005.1 15813176

[B4] Du Y. L. Chen S. F. Cheng L. Y. Shen X. L. Tian Y. Li Y. Q. (2009). Identification of a novel *Streptomyces chattanoogensis* L10 and enhancing its natamycin production by overexpressing positive regulator ScnRII. *J. Microbiol.* 47 506–513. 10.1007/s12275-009-0014-0 19763427

[B5] Du Y. L. Li S. Z. Zhou Z. Chen S. F. Fan W. M. Li Y. Q. (2011a). The pleiotropic regulator AdpAch is required for natamycin biosynthesis and morphological differentiation in *Streptomyces chattanoogensis*. *Microbiology* 157 1300–1311. 10.1099/mic.0.046607-0 21330439

[B6] Du Y. L. Shen X. L. Yu P. Bai L. Q. Li Y. Q. (2011b). Gamma-butyrolactone regulatory system of *Streptomyces chattanoogensis* links nutrient utilization, metabolism and developmental programme. *Appl. Environ. Microbiol.* 77 8415–8426. 10.1128/AEM.05898-11 21948843 PMC3233056

[B7] Gallegos M. T. Schleif R. Bairoch A. Hofmann K. Ramos J. L. (1997). Arac/XylS family of transcriptional regulators. *Microbiol. Mol. Biol. Rev.* 61 393–410. 9409145 10.1128/mmbr.61.4.393-410.1997 PMC232617

[B8] Gust B. Challis G. L. Fowler K. Kieser T. Chater K. F. (2003). PCR-targeted *Streptomyces* gene replacement identifies a protein domain needed for biosynthesis of the sesquiterpene soil odor geosmin. *Proc. Natl. Acad. Sci. U.S.A.* 100 1541–1546. 10.1073/pnas.0337542100 12563033 PMC149868

[B9] Guyet A. Gominet M. Benaroudj N. Mazodier P. (2013). Regulation of the clpP1clpP2 operon by the pleiotropic regulator AdpA in *Streptomyces lividans*. *Arch. Microbiol.* 195 831–841. 10.1007/s00203-013-0918-2 24196782

[B10] Hara H. Ohnishi Y. Horinouchi S. (2009). DNA microarray analysis of global gene regulation by A-factor in *Streptomyces griseus*. *Microbiology* 155 2197–2210. 10.1099/mic.0.027862-0 19389771

[B11] Hopwood D. A. (2007). *Streptomyces in Nature and Medicine. The Antibiotic Makers.* New York, NY: Oxford University Press Inc.

[B12] Horinouchi S. (2002). A microbial hormone, A-factor, as a master switch for morphological differentiation and secondary metabolism in *Streptomyces griseus*. *Front. Biosci.* 7 d2045–d2057. 12165483 10.2741/A897

[B13] Kato J. Y. Chi W. J. Ohnishi Y. Hong S. K. Horinouchi S. (2005a). Transcriptional control by A-factor of two trypsin genes in *streptomyces griseus*. *J. Bacteriol.* 187 286–295. 15601713 10.1128/JB.187.1.286-295.2005 PMC538825

[B14] Kato J. Y. Ohnishi Y. Horinouchi S. (2005b). Autorepression of AdpA of the AraC/XylS family, a key transcriptional activator in the A-factor regulatory cascade in *Streptomyces griseus*. *J. Mol. Biol.* 350 12–26. 15907934 10.1016/j.jmb.2005.04.058

[B15] Kieser T. Bibb M. J. Buttner M. J. Chater K. F. Hopwood D. A. (2000). *Practical Streptomyces Genetics.* Norwich: John Innes Foundation.

[B16] Komatsu M. Uchiyama T. Omura S. Cane D. E. Ikeda H. (2010). Genome-minimized *Streptomyces* host for the heterologous expression of secondary metabolism. *Proc. Natl. Acad. Sci. U.S.A.* 107 2646–2651. 10.1073/pnas.0914833107 20133795 PMC2823899

[B17] Liu G. Chater K. F. Chandra G. Niu G. Tan H. (2013). Molecular regulation of antibiotic biosynthesis in *Streptomyces*. *Microbiol. Mol. Biol. Rev.* 77 112–143. 10.1128/MMBR.00054-12 23471619 PMC3591988

[B18] López-García M. T. Santamarta I. Liras P. (2010). Morphological differentiation and clavulanic acid formation are affected in a *Streptomyces clavuligerus adpA*-deleted mutant. *Microbiology* 156 2354–2365. 10.1099/mic.0.035956-0 20447998

[B19] Macneil D. J. Klapko L. M. (1987). Transformation of *Streptomyces avermitilis* by plasmid DNA. *J. Ind. Microbiol.* 2 209–218. 10.1007/BF01569542

[B20] Mak S. Xu Y. Nodwell J. R. (2014). The expression of antibiotic resistance genes in antibiotic-producing bacteria. *Mol. Microbiol.* 93 391–402. 10.1111/mmi.12689 24964724

[B21] Mao X. M. Zhou Z. Cheng L. Y. Hou X. P. Guan W. J. Li Y. Q. (2009). Involvement of SigT and RstA in the differentiation of *Streptomyces coelicolor*. *FEBS. Lett.* 583 3145–3150. 10.1016/j.febslet.2009.09.025 19755120

[B22] McKenzie N. L. Nodwell J. R. (2007). Phosphorylated AbsA2 negatively regulates antibiotic production in *Streptomyces coelicolor* through interactions with pathway-specific regulatory gene promoters. *J. Bacteriol.* 189 5284–5292. 10.1128/JB.00305-07 17513473 PMC1951880

[B23] Moody S. C. (2014). Microbial co-culture: harnessing intermicrobial signaling for the production of novel antimicrobials. *Future Microbiol.* 9 575–578. 10.2217/fmb.14.25 24957083

[B24] Nguyen K. T. Tenor J. Stettler H. Nguyen L. T. Nguyen L. D. Thompson C. J. (2003). Colonial differentiation in *Streptomyces coelicolor* depends on translation of a specific codon within the *adpA* gene. *J. Bacteriol.* 185 7291–7296. 10.1128/JB.185.24.7291-7296.2003 14645292 PMC296263

[B25] Ohnishi Y. Kameyama S. Onaka H. Horinouchi S. (1999). The A-factor regulatory cascade leading to streptomycin production in *Streptomyces griseus*: identification of a target gene of the A-factor receptor. *Mol. Microbiol.* 34 102–111. 10.1046/j.1365-2958.1999.01579.x 10540289

[B26] Ohnishi Y. Yamazaki H. Kato J. Y. Tomono A. Horinouchi S. (2005). AdpA, a central transcriptional regulator in the A-factor regulatory cascade that leads to morphological development and secondary metabolism in *Streptomyces griseus*. *Biosci. Biotechnol. Biochem.* 69 431–439. 10.1271/bbb.69.431 15784968

[B27] Onaka H. Horinouchi S. (1997). DNA-binding activity of the A-factor receptor protein and its recognition DNA sequences. *Mol. Microbiol.* 24 991–1000. 10.1046/j.1365-2958.1997.4081772.x 9220006

[B28] Pan Y. Liu G. Yang H. Tian Y. Tan H. (2009). The pleiotropic regulator AdpA-L directly controls the pathway-specific activator of nikkomycin biosynthesis in *Streptomyces ansochromogenes*. *Mol. Microbiol.* 72 710–723. 10.1111/j.1365-2958.2009.06681.x 19400773

[B29] Retzlaff L. Distler J. (1995). The regulator of streptomycin gene expression, StrR, of *Streptomyces griseus* is a DNA binding activator protein with multiple recognition sites. *Mol. Microbiol.* 18 151–162. 10.1111/j.1365-2958.1995.mmi_18010151.x 8596455

[B30] Santos-Aberturas J. Vicente C. M. Payero T. D. Martín-Sánchez L. Cañibano C. Martín J. F. (2012). Hierarchical control on polyene macrolide biosynthesis: PimR modulates pimaricin production via the PAS-LuxR transcriptional activator PimM. *PLoS One* 7:e38536. 10.1371/journal.pone.0038536 22693644 PMC3367932

[B31] Santos-Beneit F. Rodriguez-Garcia A. Sola-Landa A. Martin J. F. (2009). Cross-talk between two global regulators in Streptomyces: PhoP and AfsR interact in the control of *afsS, pstS* and *phoRP* transcription. *Mol. Microbiol.* 72 53–68. 10.1111/j.1365-2958.2009.06624.x 19220751

[B32] Sun J. Kelemen G. H. Fernandez-Abalos J. M. Bibb M. J. (1999). Green fluorescent protein as a reporter for spatial and temporal gene expression in *Streptomyces coelicolor* A3(2). *Microbiology* 145 2221–2227. 10.1099/00221287-145-9-2221 10517575

[B33] Takano E. Chakraburtty R. Nihira T. Yamada Y. Bibb M. J. (2001). A complex role for the gamma-butyrolactone SCB1 in regulating antibiotic production in *Streptomyces coelicolor* A3(2). *Mol. Microbiol.* 41 1015–1028. 10.1046/j.1365-2958.2001.02562.x 11555283

[B34] Tan G. Y. Peng Y. Lu C. Bai L. Zhong J. J. (2015). Engineering validamycin production by tandem deletion of γ-butyrolactone receptor genes in *Streptomyces hygroscopicus* 5008. *Metab. Eng.* 28 74–81. 10.1016/j.ymben.2014.12.003 25527439

[B35] Tomono A. Tsai Y. Yamazaki H. Ohnishi Y. Horinouchi S. (2005). Transcriptional control by A-factor of strR, the pathway-specific transcriptional activator for streptomycin biosynthesis in *Streptomyces griseus*. *J. Bacteriol.* 187 5595–5604. 10.1128/JB.187.16.5595-5604.2005 16077104 PMC1196073

[B36] van Wezel G. P. McDowall K. J. (2011). The regulation of the secondary metabolism of Streptomyces: new links and experimental advances. *Nat. Prod. Rep.* 28 1311–1333. 10.1039/c1np00003a 21611665

[B37] Yamazaki H. Ohnishi Y. Horinouchi S. (2003a). Transcriptional switch on of ssgA by A-factor, which is essential for spore septum formation in *Streptomyces griseus*. *J. Bacteriol.* 185 1273–1283. 12562798 10.1128/JB.185.4.1273-1283.2003 PMC142869

[B38] Yamazaki H. Tomono A. Ohnishi Y. Horinouchi S. (2004). DNA-binding specificity of AdpA, a transcriptional activator in the A-factor regulatory cascade in *Streptomyces griseus*. *Mol. Microbiol.* 53 555–572. 10.1111/j.1365-2958.2004.04153.x 15228534

[B39] Yu Z. Zhu H. Dang F. Zhang W. Qin Z. Yang S. (2012). Differential regulation of antibiotic biosynthesis by DraR-K, a novel two-component system in *Streptomyces coelicolor*. *Mol. Microbiol.* 85 535–556. 10.1111/j.1365-2958.2012.08126.x 22676800

