# Supplementary material for: Bidirectional Regulation of AdpAch in Controlling the Expression of scnRI and scnRII in the Natamycin Biosynthesis of Streptomyces chattanoogensis L10
Source: Front Microbiol. 2018 Mar 2;9:316. doi: 10.3389/fmicb.2018.00316 (PMC5840217; doi:10.3389/fmicb.2018.00316)
Supplement: Supplementary file 1 [file Table_1.doc]

**Supplementary Information**

**Table S1. Oligonucleotides used in this study.**

| Primer and purpose Sequence |
| --- |
| **For EMSA analysis**  RI-RII-F ACGTTTTATCAAGGCTCG  RI-RII-R CGCAGATGAGAAACAT  Site A-F GCGGATATGGCTGACGGACGATTTCGAGCTGACTGTTCTCCTACCGA  Site A-R CGGTAGGAGAACAGTCAGCTCGAAATCGTCCGTCAGCCATATCCGCA  Site mA-F GCGGATAGATATCACGGACGATTTCGAGCGATATCTTCTCCTACCGA  Site mA-R CGGTAGGAGAAGATATCGCTCGAAATCGTCCGTGATATCTATCCGCA  Site B-F TCCCCTGCGGATCTTGACATAGAGAAAGCACA  Site B-R GTGCTTTCTCTATGTCAAGATCCGCAGGGGAA  Site mB-F TCCCCTGCGGATCGATATCTAGAGAAAGCACA  Site mB-R GTGCTTTCTCTAGATATCGATCCGCAGGGGAA  Site C-F ATTGGCAAGAAAGCGGCAGGTGTTCGGCAAGGATTTCGACAAGGGA  Site C-R CCCTTGTCGAAATCCTTGCCGAACACCTGCCGCTTTCTTGCCAATA  Site mC-F ATGATATCGAAAGCGGCAGGTGTTCGGCAAGGATTTCGACAAGGGA  Site mC-R CCCTTGTCGAAATCCTTGCCGAACACCTGCCGCTTTCGATATCATA  Site D-F GCAAGGATTTCGACAAGGGGTCCGGAAAACGATA  Site D-R ATCGTTTTCCGGACCCCTTGTCGAAATCCTTGCA  Site mD-F GCAAGGATTGATATCAGGGATATCGAAAACGATA  Site mD-R ATCGTTTTCGATATCCCTGATATCAATCCTTGCA  Site E-F AAGGGGCCGCCAGTGGCTCGACCAGGACCCCGCAAGGGCA  Site E-R GCCCTTGCGGGGTCCTGGTCGAGCCACTGGCGGCCCCTTA  Site mE-F AAGGGATATCCAGGATATCGACCAGGACCCCGCAAGGGCA  Site mE-R GCCCTTGCGGGGTCCTGGTCGATATCCTGGATATCCCTTA  Site F-F TTCACCGCCGCTCGCCAGCCTCCGAATAGATTTCGA  Site F-R CGAAATCTATTCGGAGGCTGGCGAGCGGCGGTGAAA  Site mF-F TTCACCGCCGCTCGCCAGCCTCCGAATAGATTTCGA  Site mF-R CGAAATCTATTCGGAGGCTGGCGAGCGGCGGTGAAA  **For *xylE* fusions with mutated sites**  *xylE*-F AGCCATATGATGAACAAAGGTGTAATG  *xylE*-R AAGGCGGCCGCTCAGGTGAGCACGGTCATGAA  Site mA-F GCGGATATGGCTGACGGACGATTTCGAGCTGACTGTTCTCCTACCGA  Site mA-R CGGTAGGAGAACAGTCAGCTCGAAATCGTCCGTCAGCCATATCCGCA  Site mB-F TCCCCTGCGGATCTTGACATAGAGAAAGCACA  Site mB-R GTGCTTTCTCTATGTCAAGATCCGCAGGGGAA  Site mC-F ATTGGCAAGAAAGCGGCAGGTGTTCGGCAAGGATTTCGACAAGGGA  Site mC-R CCCTTGTCGAAATCCTTGCCGAACACCTGCCGCTTTCTTGCCAATA  Site mD-F GCAAGGATTTCGACAAGGGGTCCGGAAAACGATA  Site mD-R ATCGTTTTCCGGACCCCTTGTCGAAATCCTTGCA  Site mE-F AAGGGGCCGCCAGTGGCTCGACCAGGACCCCGCAAGGGCA  Site mE-R GCCCTTGCGGGGTCCTGGTCGAGCCACTGGCGGCCCCTTA  Site mF-F TTCACCGCCGCTCGCCAGCCTCCGAATAGATTTCGA  Site mF-R CGAAATCTATTCGGAGGCTGGCGAGCGGCGGTGAAA  **For construction of mutated sites in vivo**  scnRI-F CCATCGTCGTGATCTCCT  scnRII-R GACGAATCGCTGATGCTT  Site-A-mut-F GAGAAGATATCGCTCGAAATCGTCCGTGATATCTATCCGCGAGAACCATGG  Site-A-mut-R GCGGATAGATATCACGGACGATTTCGAGCGATATCTTCTCCTA  Site-B-mut-F GCTTTCTCTAGATATCGATCCGCAGGGGACGGTAGGAG  Site-B-mut-R CCTGCGGATCGATATCTAGAGAAAGCACCTTCCAGGGT  Site-C-mut-F TGCCGCTTTCGATATCATCGATGGGCATCCACCCTGGA  Site-C-mut-R GCCCATCGATGATATCGAAAGCGGCAGGTGTTCGGCAA  Site-D-mut-F CCCCTTGCCATCGCCTGCCATCGTTTTCGATATCCCTGATATCAATC  Site-D-mut-R AAGCGGCAGGTGTTCGGCAAGGATTGATATCAGGGATATCGAAAAC  Site-E-mut-F TCATCGCCCTTGCGGGGTCCTGGTCGATATCCTGGGATATCCTTGCC  Site-E-mut-R GAAAACGATGGCAGGCGATGGCAAGGATATCCCAGGATATCGACCAGGA  Site-F-mut-F CGAAATCTATGATATCGCTGGCGAGCGGCGGTGAAGGC  Site-F-mut-R GCTCGCCAGCGATATCATAGATTTCGCCCTCAAGTTCT |
